# Phenotypes and genetic etiology of spontaneous polycystic kidney and liver disease in cynomolgus monkey

**DOI:** 10.3389/fvets.2023.1106016

**Published:** 2023-02-16

**Authors:** Ruo Wu, Bing Bai, Feng Li, Raoxian Bai, Yan Zhuo, Zhengna Zhu, Rongfang Jia, Shangang Li, Yongchang Chen, Xiaoping Lan

**Affiliations:** ^1^State Key Laboratory of Primate Biomedical Research and Institute of Primate Translational Medicine, Kunming University of Science and Technology, Kunming, China; ^2^Yunnan Key Laboratory of Primate Biomedical Research, Kunming, China; ^3^Kunming Biomed International, Kunming, China; ^4^Molecular Diagnostic Laboratory, Shanghai Children's Hospital, School of Medicine, Shanghai Jiao Tong University, Shanghai, China

**Keywords:** polycystic kidney disease, polycystic liver disease, whole-genome sequencing, cynomolgus monkey, phenotype, genetic etiology

## Abstract

**Introduction:**

Polycystic kidney disease (PKD) is a common autosomal dominant or recessive genetic disease, often accompanied by polycystic liver disease (PLD). Many cases of PKD in animals have been reported. However, little is known about the genes that cause PKD in animals.

**Methods:**

In this study, we evaluated the clinical phenotypes of PKD in two spontaneously aged cynomolgus monkeys and explored the genetic etiology using whole-genome sequencing (WGS). Ultrasonic and histological consequences were further investigated in PKD- and PLD-affected monkeys.

**Results:**

The results indicated that the kidneys of the two monkeys had varying degrees of cystic changes, and the renal cortex was thinned and accompanied by fluid accumulation. As for hepatopathy, inflammatory cell infiltration, cystic effusion, steatosis of hepatocytes, and pseudo-lobular were found. Based on WGS results, the variants of PKD1:(XM_015442355: c.1144G>C p. E382Q) and GANAB: (NM_001285075.1: c.2708T>C/p. V903A) are predicted to be likely pathogenic heterozygous mutations in PKD- and PLD-affected monkeys.

**Discussion:**

Our study suggests that the cynomolgus monkey PKD and PLD phenotypes are very similar to those in humans, and are probably caused by pathogenic genes homologous to humans. The results indicate that cynomolgus monkeys can be used as the most appropriate animal model for human PKD pathogenesis research and therapeutic drug screening.

## 1. Introduction

Autosomal dominant polycystic kidney disease (ADPKD) is a chronic progressive kidney disease that affects ~1 in 1,000 adults, half of whom progress to end-stage renal disease (ESRD) after middle age (1); autosomal recessive polycystic kidney disease (ARPKD) is commonly diagnosed in childhood or even earlier, with a prevalence of ~1:20,000 (2). Autosomal dominant polycystic liver disease (ADPLD) is characterized by PLD and includes severe, symptomatic disease identical to ADPKD but without (or only occasionally with) renal cysts. Metabolic dysfunction may occur in the liver and kidneys of patients with PKD and PLD (3).

Approximately 85% of ADPKD cases are caused by mutations in *PKD1* (located on chromosome 16p13.3), and the remaining 15% of the clinically defined population are affected by mutations in *PKD2* (located on chromosome 4p21). In 66.6% of patients with ADPKD, multiple types of mutations in *PKD1* and *PKD2* (non-sense, frameshift, canonical splicing, and large rearrangements) result in structural disruption and loss of protein function, and these mutations are generally considered pathogenic. However, in the remaining 33.3% of patients with ADPKD, the pathogenic variant cannot be identified by sequencing. According to the sequencing results, in-frame indels or atypical splicing are frequently found in *PKD1* and *PKD2*, but these types of mutations do not alter the gene reading frame. Therefore, their pathogenicity is less clear and requires further analysis. Gene mutations in the *PKD1* and *PKD2* genes are complex and diverse (4). Clinical variability is closely related to the gene and its mutation types. For example, the median age at the onset of ESRD in most patients with PKD2 mutations is 79 years, which is 20 years later than that in patients with PKD1 mutations, and the prognosis of the disease is also better in the former (5). Human pathogenic ADPKD mutations have been compiled in a central database (6). On the basis of the case mutation statistics reports, different mutation types have been found in almost full-length *PKD1* and *PKD2* genes, and no obvious clustering was observed (7).

In the past few decades, more than 30 genes responsible for human PKD and PLD have been identified using genome-sequencing technology (8, 9). The gene products responsible for PKD and PLD are distributed in the primary cilia and are thought to be key proteins controlling Ca^2+^ cycling and intercellular signaling. In PKD research, the key roles of signaling pathways, including MAPK, MTOR, and PPAR-γ, have been identified through animal models (10–12). The mouse model is currently the most used animal model of PKD and PLD; however, mice and humans differ dramatically in both their physiological and genetic structures.

In 2019, Tomoyuki Tsukiyama et al. (13) successfully used CRISPR/Cas9 to generate cynomolgus monkey ADPKD models with *PKD1* mutations. Unlike mice, heterozygous *PKD1* mutant cynomolgus monkey models or patients usually have a polycystic kidney phenotype. The PKD cynomolgus monkey models recapitulate patients with ADPKD, indicating that the cynomolgus monkey is the most appropriate animal model for human ADPKD treatment strategy selection and drug development research. At present, there are few reports on spontaneous ADPKD monkeys. As early as 1984, Kessler (14) reported a case of polycystic kidney in a rhesus monkey. In 1990, Sakakibara (15) reported a spontaneous case of polycystic kidney in cynomolgus monkeys, although there are few reports based on detailed information on PKD-related pathogenic genes in animals. Herein, we report the phenotypes of two cases of fibrocystic polycystic kidney in cynomolgus monkeys, accompanied by a severely polycystic liver in one case. In addition, we performed WGS analysis on the two PKD-/PLD-affected monkeys to identify the mutations in the disease-causing genes.

## 2. Materials and methods

### 2.1. Animals

The animals were housed in temperature-controlled chambers and had free access to water and food. Animal facilities in this study were approved by AAALAC International, and all animal handling procedures were approved in advance by the Institutional Animal Care and Use Committee of Kunming Biomed International (license number: KBI K001116020-01, 01). Kunming Biomed International is a professionally qualified non-human primate scientific research institution, and some monkeys from animal rescue stations or cynomolgus monkeys with scientific research needs are bred at the base every year. KBI has primate breeding facilities that meet the standards of the Association for Evaluation and Certification of Laboratory Animal Management (AAALAC). Two ADPKD cynomolgus monkeys were used for our experiments, both living in Yunnan Province, China, who had never been used in any drug or medical experiments until their death.

### 2.2. Histology and H&E staining

Due to improper preservation of the specimen, after dissection, the livers and kidneys of the PKD- and PLD-affected cynomolgus monkeys were used for histology analysis. The cross sections were equilibrated at room temperature for 15 min, and the nuclei were dyed with Harris' hematoxylin for 5 min. The slices were rinsed under running water for 5 min, and 1% eosin staining was performed for 5 min. After dehydration in a gradient ethanol bath and washing with xylene, the patches were sealed with neutral gum and observed under the bright field of a microscope (Leica, Germany).

### 2.3. Ultrasonography

The ultrasound procedure was performed using a GE LOGIQ P5 ultrasound imaging system after the onset of short-acting Zoletil anesthetic (0.1 mg/kg i.m.); then, the animal was safely secured to the examination table. Abdominal ultrasonography of monkey 010139 affected by PKD and PLD was performed after it exhibited depression and poor appetite. The other PKD-affected monkey was diagnosed during necropsy; therefore, no relevant ultrasonography results were available. Animal IDs and health status were confirmed before biopsy procedures. Animals were anesthetized with ketamine, 10 mg/kg i.m., and hair on the area of skin that covered the right chest and the liver lobe was removed using a razor. The imaging probe was placed on the abdomen. Ultrasonographic examinations were conducted as previously described (16). The liver and other organs were imaged transcutaneously using the GE LOGIQ P5 ultrasound imaging system, with 10 L and 4°C probes (GE Healthcare, USA).

### 2.4. Blood/serum biochemical assays

Before the ADPKD monkeys died, we collected serum by venipuncture of the saphenous vein using a 5-mm animal prick needle (Goldenrod Animal Lancet, Braintree Scientific). Then, 2 ml of blood was collected in an anticoagulant-free blood collection tube (BD vacutainer, SST II Advance). The monkeys were carefully monitored for 30 min following blood withdrawal to watch for signs of distress. An additional 0.5 ml of blood was collected into an EDTA-K2 tube. Biochemical index levels were determined by Kunming Biomed International Laboratory using a Roche Cobas C501 (Roche, Germany). Hemocytometry was performed on a Sysmex XT-2000i (Sysmex, Japan).

### 2.5. Whole-genome sequencing

After the ADPKD monkeys died, genomic DNA was extracted from the livers and/or kidneys using the protocol of the Wizard Genomic DNA Purification Kit. DNA concentration was measured with NanoDrop2000 (Thermo Scientific, Germany). Whole-genome resequencing and bioinformatics analysis were performed according to the whole-genome resequencing workflow by the BGI Shenzhen Corporation under the contract terms of project number F20FTSCCKF0459_MONldhR. The qualified DNA samples were randomly fragmented by Covaris, and the fragments were collected by magnetic beads after sample quality control. Adenines were added to the 3′-end of end-repaired DNA fragments before adaptor ligation. The ligation products were then cyclized and amplified using linear isothermal rolling-circle replication and DNA nanoball technology. BGISEQ was used to sequence these DNA libraries. Bioinformatics analysis was performed with the sequencing raw data generated by the sequencing pipeline. In the raw data, low-quality reads were removed first. Then, the Burrows–Wheeler Aligner was used to align the reads to the reference sequence. Alignment information was stored in BAM format files, which were further processed in the following steps: sort by samtools, filter mapping quality, and label duplicate reads. GATK (https://www.broadinstitute.org/gatk/) was used to detect single-nucleotide polymorphisms (SNPs) and small insertions and deletions (Indels) in the post-processed BAM files. Break Dancer (http://breakdancer.sourceforge.net/) was used for structural variants (SVs) calling and Control-FREEC (http://boevalab.inf.ethz.ch/FREEC/index.html#introduction) for copy number variants (CNVs) calling. All variants were annotated by Annovar (17, 18). Mutation pathogenicity prediction analysis was performed on the Mutation Taster website (https://rddc.tsinghua-gd.org/). The cynomolgus monkey whole-genome data reference version is GCF_000364345.1_Macaca_fascicularis_5.0.

### 2.6. Primer design and PCR reaction conditions

Primers for *PKD1* and *PKD2* target genes were designed using the Premier 6.0 software. The primer sequences were as follows: PKD1-F2: GTGGTGGAGATGGACGTAGAGT; PKD1-R2: CGGAAATAGGGCAGGATTAGCG; PKD1-F3: ACACA GTGGAGCATGTGTACC; PKD1-R3: GACCTTGATGTCCGTGAC CA; PKD1-F4: CGAGTCACCATCACGGATGGT; PKD1-R4: ACCCAGGCAGGCACTATGAGA; PKD2-F3: AATGGTGGTGGAGATGG ACGTA; PKD2-R3: CCCCAGATGTACTCTTTCACTCTAA; GANAB-F1: GAATGTATGAAGGATGACCCAA; GANAB-R: CGCAGGTGAATACTCCAATC. The reaction conditions and PCR system were as follows: 1.5 μl of DNA template (200 ng/μl), 1 μl of each upstream and downstream primer (10 μmol/l), 2 μl of dNTP mix, 1 μl of PrimeSTAR GXL DNA polymerase (TAKARA), 10 μl of 5× PrimeSTAR GXL buffer Mg^2+^, 4 μl of dNTP mixture, and up to 50 μl of nucleic acid-free water; amplification of the PCR reaction: 3 min predenaturation at 95°C, 30 s denaturation at 94°C, 15 s annealing temperature, 30 s extension at 72°C, 35 cycles, 5 min extension at 72°C, and storage at 4°C. After electrophoresis on a 1% agarose gel at 110 V for 30 min and staining, PCR products were detected with a gel imager. Sequencing reactions were prepared using a BigDye^®^ Terminator kit (Applied Biosystems), and reaction products were sequenced using the ABI 3500Dx Genetic Analyzer (Applied Biosystems, Forster City, USA). Sanger sequencing was performed by Tsingke Biotechnology Co., Ltd.

### 2.7. RDDC: Variant pathogenicity prediction of disease-causing genes

We screened for variants where the WGS and Sanger sequencing results were completely consistent and clarified the mutation information of these sites in the human genome. The pathology prediction tool on the website (https://rddc.tsinghua-gd.org/tools) is based on a machine-learning approach that uses the XGBoost classifier to predict pathogenicity. The predicted outcome is a number between 0 and 1, with a higher number (closer to 1) indicating a higher probability that the mutation at the locus is pathogenic. Encoded values and qualitative prediction are as follows: 0–0.02: Benign; 0.02–0.1: Likely Benign; 0.1–0.8: Likely Pathogenic; and 0.8–1: Pathogenic.

## 3. Results

### 3.1. Phenotype

**Figure 1:**
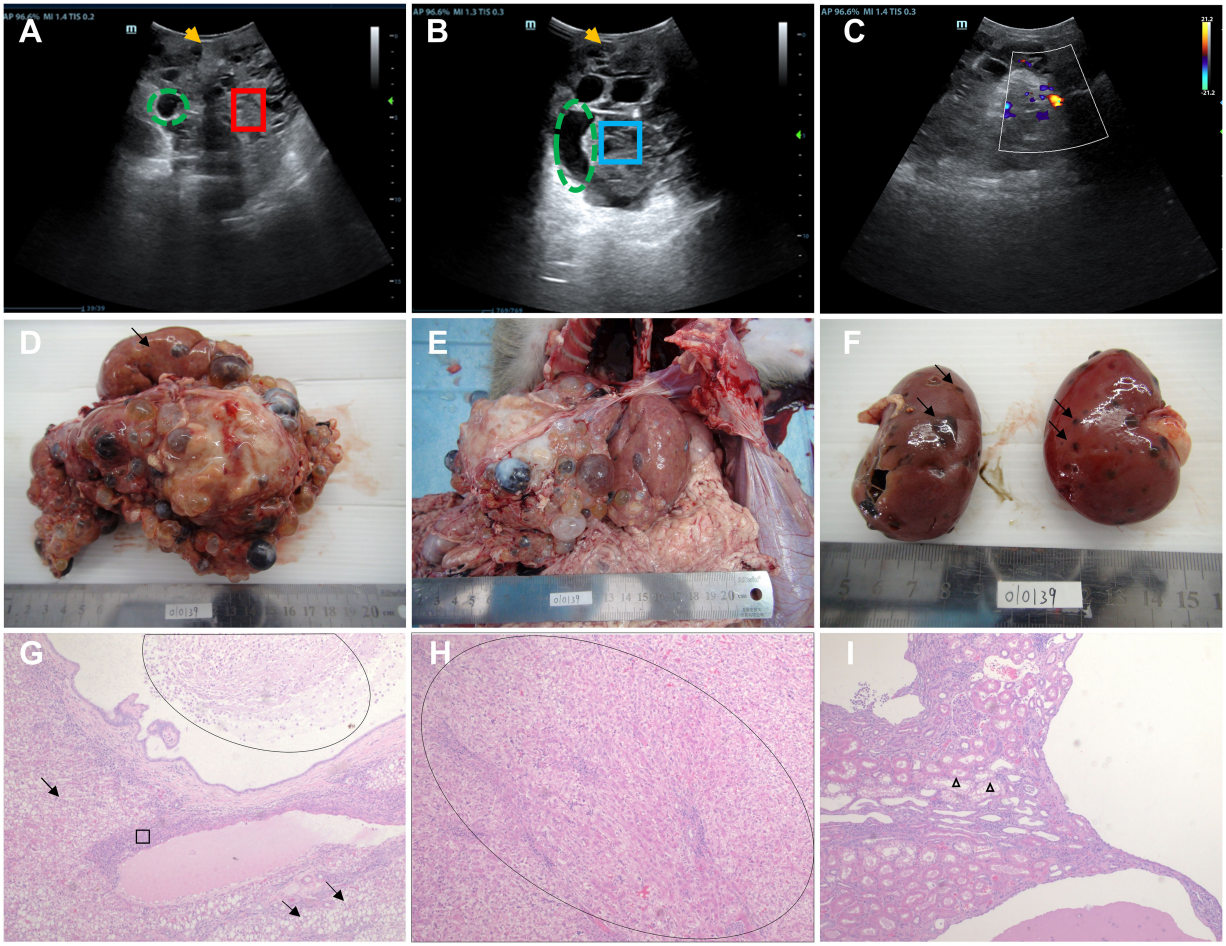
Ultrasound imaging, macroscopic specimen, and histopathological features of monkey 010139. **(A)** Ultrasound examination showed 010139 liver enlargement, an uneven echo of the liver capsule, and an unclear shape (yellow arrow). The normal liver structure has disappeared, and the liver parenchyma (red box) shows multiple cyst-like structures of varying sizes (green dotted circle). **(B)** Ultrasonography showed an uneven echo of the 010139 liver capsule and unclear shape (yellow arrow). Several circular and elliptical cystic structures are visible (green dashed circles). The wall is thin and smooth, and the large sac-like structure forms many silencing areas (blue frames) with neat edges. **(C)** Color Doppler flow imaging indicates that no blood flow signal was detected in each of the liquid dark areas. **(D)** Liver, size: 17 × 15 × 10 cm. Most of the normal liver tissue (black arrow) had disappeared, and all liver segments (>100 cysts) had small- to medium-sized liver cysts (diameter <0.5–2 cm), filled with pale yellow or brown tissue exudate. **(E)** The morphology and structure of normal liver tissue had disappeared, the boundary was unclear, and the liver expanded to the pelvic cavity. **(F)** Kidney, size: 6 × 3.5 × 3 cm. Some small cysts formed on the surface (black arrow) of the renal cortex with necrotic rupture of the cyst wall. Hematoxylin–eosin staining (H&E) images of 010139's liver are shown in **(G, H)**. **(G)** Diffuse steatosis of hepatocytes (black arrow), multiple cystic cavities, eosinophilic secretions and macrophages (oval circles), pericystic fibrosis, and interstitial lymphocytic infiltration (ellipse). **(H)** Pseudo-lobular formation is shown in the oval area. **(I)** Kidney tubular dilation (triangle), eosinophilic changes in epithelial cells, foamy changes in some epithelial cells, no normal glomerular structure, interstitial lymphocyte infiltration, multiple cystic cavities, and eosinophilic discharge in some cysts. Original magnification: 4×.

Diseased monkey 1: We investigated a 17-year-old male PKD- and PLD-affected cynomolgus monkey (Monkey 010139). Veterinarians observed that the monkey's abdomen was bulging and that it exhibited depression and poor appetite. Ultrasonography of 010139 showed an enlarged liver with the presence of multiple cysts that were not clearly separated from the kidneys (Figures 1A–C). We analyzed the serum biochemical indexes of the male cynomolgus monkeys. The serum index results were as follows: creatinine 47 μmol/L, blood urea nitrogen 6.7 mmol/L, aspartate aminotransferase 38 U/L, alanine aminotransferase 27.2 U/L, albumin 15.2 g/L, alkaline phosphatase 4,719 U/L, total protein 53.7 g/L, Na^+^ 136 mmol/L, K^+^ 4.55 mmol/L, CL^−^ 98.4 mmol/L, globulin 38.5 g/L, A/G 0.4, and ketone 2,249 μmol/L. Routine blood examination showed the following values: leukocyte 20.5 × 10^9^/L, erythrocyte 6.01 × 10^12^/L, hemoglobin 119 g/L, hematocrit 38.9%, platelets 364 × 10^9^/L, neutrophils 69.9%, and lymphocytes 24%. Reference values of clinical pathology parameters in cynomolgus monkeys used in this study are listed in Supplementary Table S1 (19, 20). The normal leukocyte range in the blood of cynomolgus monkeys is (11.15 ± 2.85) × 10^9^/L, and the normal value of ketone bodies in the serum of cynomolgus monkeys should be lower than 0.6 mmol/L (19, 20). The above results showed that 010139 had inflammatory infection and ketosis without liver and kidney dysfunction. After a definitive diagnosis of inflammatory infection and ketosis, the first line of treatment is rapid anti-inflammatory therapy and nutritional support. Accordingly, veterinarians administered an intravenous drip comprising ceftriaxone sodium (80 mg/kg) anti-inflammatory therapy, vitamin supplementation, and energy support with rehydration salts.

We performed an autopsy on Monkey 010139 when it died 1 month later despite active treatment, due to refractory infection. Normal liver tissue (indicated by black arrows) had mostly disappeared (Figure 1D), and there were small- to medium-sized liver cysts (diameter between 0.5 and 2 cm) in all liver segments (>100 cysts). Multicystic structures filled with tissue exudate were observed, some of which were light yellow and some were brown (Figures 1D, E). Most of the kidney surface cortex was relatively smooth, with around 12 vesicles; multiple vesicles had spontaneously ruptured, and tissue necrosis was observed (Figure 1F). The results of hematoxylin–eosin staining (H&E) of liver tissue showed diffuse steatosis of hepatocytes, a liver cyst wall lined with a single layer of columnar epithelium, eosinophilic secretions and macrophages in the lumen, pericystic tissue fibrosis, and interstitial lymphocyte infiltration (Figure 1G). The normal hepatic lobular structure was destroyed, and the hepatocyte-regenerated nodules were divided and wrapped into round or oval pseudo-lobules of varying sizes by extensively proliferating fibrous tissue (Figure 1H). Histopathological features of kidney cystic tissue are shown, including tubular dilation, eosinophilic changes in epithelial cells, and foam-like changes in some epithelial cells (Figure 1I). No normal glomerular structure was observed, and interstitial lymphocyte infiltration, multiple cystic cavities, and eosinophilic secretions were seen in some cystic cavities.

Diseased monkey 2: A 20-year-old PKD-affected monkey (Monkey 993563) was diagnosed during the process of dissection after it died spontaneously. A few renal cysts existed in bilateral kidneys (Supplementary Figure S1), and there were no obvious abnormal changes in other organs. Results related to pathological slide analysis were not collected due to improper preservation of the specimen. Neither blood cell analysis nor serum biochemical index analysis indicated abnormalities before it died. The results of serum biochemical indexes were as follows: creatinine 87 μmol/L, blood urea nitrogen 6.5 mmol/L, aspartate aminotransferase 26 U/L, alanine aminotransferase 25.3 U/L, glucose 154 mg/dl, glycated hemoglobin% 4.7, high-density lipoprotein 0.7 mmol/L, triglycerides 1.16 mmol/L, cholesterol 1.4 mmol/L, very low-density lipoprotein 0.53 mmol/L, and low-density lipoprotein cholesterol 30.6 mmol/L. Blood cell tests indicated the following values: leukocyte 9.65 × 10^9^/L, erythrocyte 6.91 × 10^12^/L, hemoglobin 168 g/L, hematocrit 55.7%, platelets 394 × 10^9^/L, neutrophils 35.4%, and lymphocytes 53.4%. None of these indicators were higher than the normal values.

### 3.2. Identifying private candidate variants

When analyzing the WGS data, autosomal recessive and dominant modes of inheritance were considered. In accordance with previous literature reports, our analysis focused on the WGS data of 31 autosomal recessive and/or dominant PLD- and PKD-related genes (PKD1, PKD2, PKHD1, HNF1B, ALMS1, TSC1, TSC2, TCTN2, B9D1, B9D2, OFD1, MKS1, DYNC2H1, TTC21B, TMEM216, TMEM67, TMEM231, CEP290, ROGRIPIL, CC2D2A, NEK1, GANAB, PRKCSH, UMOD, MUC1, SEC63, LRP5L, DZIP1L, VHL, IFT80, and DNAJB11) (8, 9). Filtering for private variants present as homozygous or heterozygous in two affected monkeys and absent in the genomes of 11 wild-type cynomolgus monkeys revealed no variants shared across all affected monkeys. The WGS data of 11 wild-type cynomolgus monkeys were obtained from literature reports by Wang et al. (21). All variants that refer to indels and SNPs per genome after annotation were compiled and are shown in Table 1. Filtering each case separately for private variants produced candidate protein-changing variants with dominant inheritance in four positions. There are no SV CNVs found in these genes related to PKD and PLD across the WGS data for the two affected monkeys.

Table 1The number of private variants divided into classes based on predicted effects.

**Table:** 

**Monkey**	**Total indels in the whole genome**	**Filtered indels**	**Genes with private variants after filtering and absent in the genomes of the 11 comparison cohorts**						
			**Filtered indels in key genes**			**Filtered indels of key genes' exons**			
			**Total**	**Intronic**	**Intergenic**	**Total**	**frameshift**	**Non-frameshift**	**Stopgain**
010139	16251616	3535084	918	588	322	8	7	1	0
993563	15479996	2591003	688	400	257	11	8	1	2

**Table:** 

	**Total SNPs in the Whole Genome**	**Filtered SNPs**	**Filtered SNPs in key genes**			**Filtered SNPs of key genes' exons**				
			**Total**	**Intronic**	**Intergenic**	**Total**	**Non-synony** **mous**	**Stopgain**	**Stoploss**	**Splicing**
010139	16251616	3535084	5831	3582	2166	83	79	2	2	0
993563	15479996	2591003	4154	2484	1615	55	52	3	0	0

In PKD- and PLD-affected monkey 010139, heterozygous missense variants were found in exons of PKD2, GANAB, and UMOD genes. Detected variants include PKD2: (XM_005555387.2: c.452C>T/p. P151L); GANAB: (NM_001285075.1: c.2708T>C/p. V903A); UMOD: (XM_005591391.1: c.970A>T: p. T324S); and UMOD: (XM_005591391.1: c.499A>G: p. T167A), identified by Sanger sequencing. The mutation status of these loci is consistent with the results of next-generation sequencing. The GANAB gene located on chromosome 11q12.3 encodes the alpha subunit of glucosidase II and is a member of the glycosyl hydrolase 31 family of proteins.

**Figure 2:**
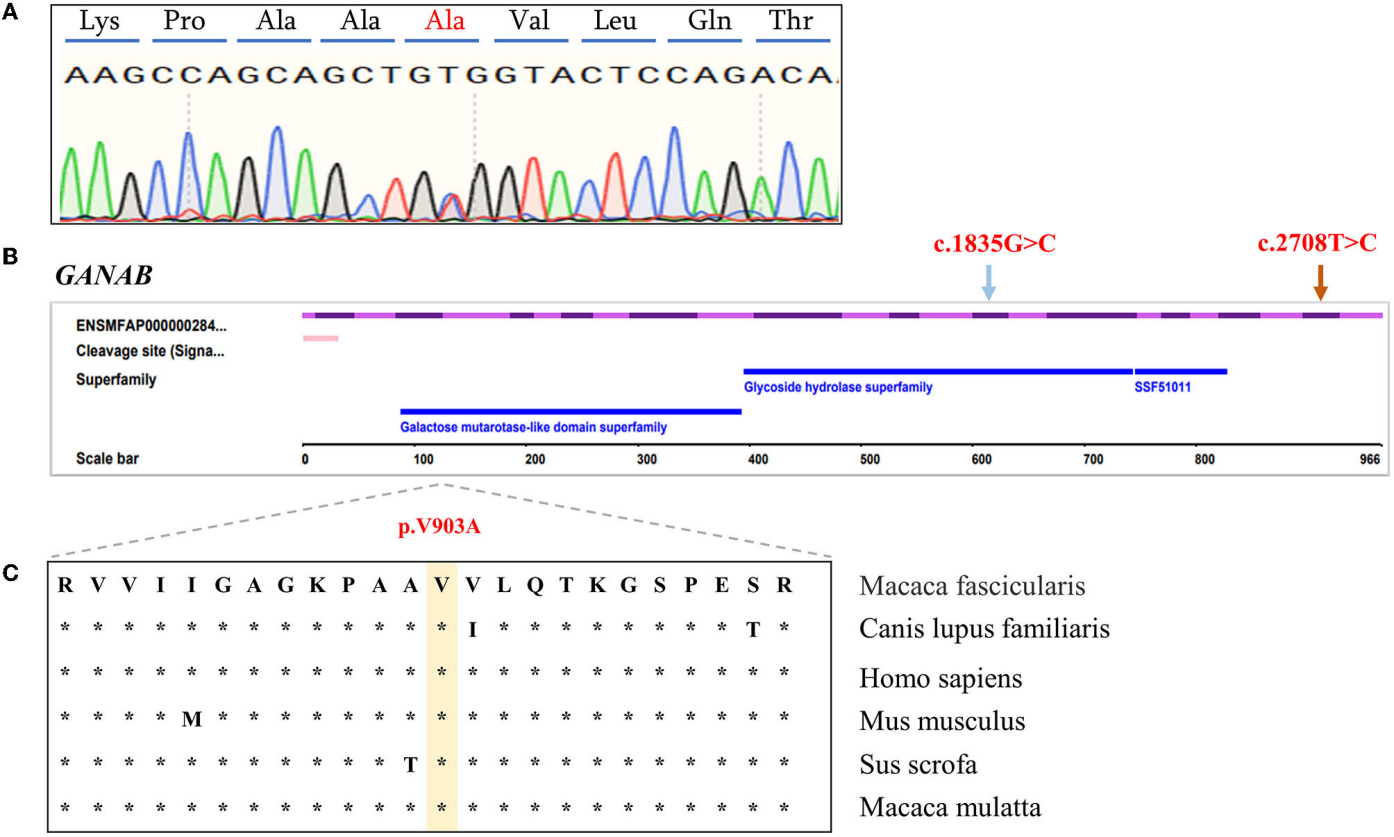
Characterization of *GANAB* functional candidate variants identified in PKD- and PLD-affected monkeys. **(A)** Sanger sequencing peak plot of missense variants in *GANAB*. **(B)** Schematic representation of *GANAB* indicating the c.2708T>C heterozygous missense candidate variant in exon 23 (orange arrow) and the previously described human variant in exon 16 (blue arrow). **(C)** Comparative analysis of amino acids at candidate positions of GANAB in multiple species.

We compared the conservation of amino acids at the mutation site across species, GANAB: (NM_001285075.1: c.2708T>C/p. V903A) is highly conserved among multiple species (Figure 2), while the variant in PKD2: (XM_005555387.2: c.452C>T/p. P151L) is leucine in mice, which is inconsistent with the corresponding amino acid in humans and monkeys (Supplementary Figure S2). Based on the conservation analysis of the position of the variants between the cynomolgus monkey and the human genome, we performed pathogenicity prediction analysis of highly conserved locations across species. The prediction analysis method is published in the database website developed by the South China Rare Disease Data Center (RDDC) (https://rddc.tsinghua-gd.org/manual/patho-predic). Encoded value and qualitative prediction are as follows: 0–0.02: Benign; 0.02–0.1: Likely Benign; 0.1–0.8: Likely Pathogenic; and 0.8–1: Pathogenic. Two variants in PKD2: (XM_005555387.2: c.452C>T/p. P151L) and GANAB: (NM_001285075.1: c.2708T>C/p. V903A) were likely to cause severe disease, with pathogenic probabilities of 0.32 and 0.16, respectively. However, variants in the UMOD gene were analyzed as benign.

**Figure 3:**
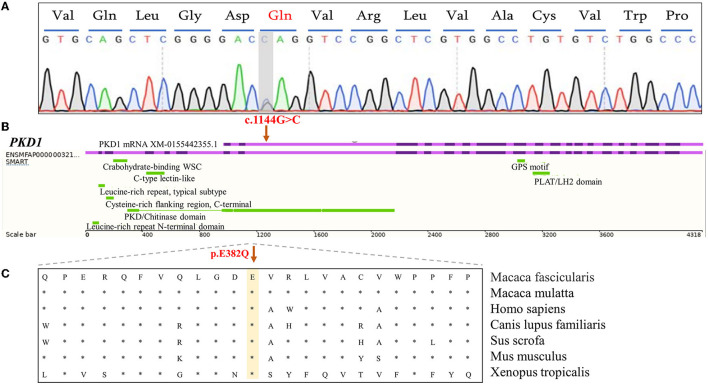
Analysis of functional candidate variants identified in *PKD1* in PKD-affected cynomolgus monkey 993563. **(A)** Forward-strand Sanger sequencing analysis of exon 4 of the *PKD1* gene in monkey 993563. The positions of mutations are indicated by gray bars. **(B)** Schematic representation of the *PKD1* gene showing the c.G1144C variant in exon 4 (purple). The corresponding cDNA position of the important domain of PKD-1 protein. **(C)** A cross-species conservation alignment (khaki) of the monkey PKD-related missense variants identified here.

In the PKD-affected monkey 993563, two missense variants in exon 4 of the polycystin-1 (*PKD1*) gene were discovered. The variants in *PKD1*: (XM_015442355.1: c.1144G>C/ p.E382Q) and *PKD1*: (XM_015442355.1: c.2488A>G/ p. S830G) affect amino acid residues 382 and 830 in PKD1, with moderate effects on the resulting protein. Missense variants affect the PKD/chitinase domain of *PKD1*, which typically consists of the *PKD1*-IgG motif. Humans have a similar missense variant in exon 4 (Figure 3). A final missense variant in exon 13 of the *PKD1*: (XM_015442355.1: c. 5530G>T/ p. A1884S) was found. Amino acid conservation analysis between species at the mutation position and mutation pathogenicity prediction analysis on the RDDC website suggested that *PKD1*: (XM_015442355.1: c.1144G>C/ p.E382Q) is a pathogenic mutation of 0.14. In humans, PKD caused by missense variants has been reported in many patients (9). The other variant of the *PKD1* gene is thought to be benign. Information on the pathogenic loci of the two PKD-/PLD-affected monkeys implicated in PKD and PLD is summarized in Table 2.

**Table 2:** Evaluation of the pathogenic potential of variants in PKD- and PLD-affected monkeys.

**Monkey**	**Gene**	**Transcript ID**	**Exon**	**cDNA change**	**Protein change**	**Consistent with human**	**Pathogenicity predict (RDDC)**	**Pathogenicity score (RDDC)**
010139	*PKD2*	XM_005555387.2	1	c.452C>T	p.P151L	Yes	Likely Pathogenic	0.051
010139	*GANAB*	XM_005577578.2	23	c.2708T>C	p.V903A	Yes	Likely Pathogenic	0.16
010139	*UMOD*	XM_005591392.2	3	c.499A>G	p.T167A	Yes	Benign	0.0027
010139	*UMOD*	XM_005591392.2	4	c.970A>T	p.T324S	Yes	Benign	0.0013
993563	*PKD1*	XM_015442355.1	4	c.1144G>C	p.E382Q	Yes	Likely Pathogenic	0.14
993563	*PKD1*	XM_015442355.1	13	c.5530G>T	p.A1844S	Yes	Benign	0.0078

## 4. Discussion

Using ultrasound imaging, macroscopic specimens, and pathological analyses, two cynomolgus monkeys were diagnosed with PKD with or without PLD, similar to humans. The two cases of PKD monkeys reported in this article were spontaneous, and it was not possible to trace the case family members and the onset genetic background. Individual cases were sequenced to investigate the underlying genetic characteristics. We found no shared variants between the two affected monkeys and the 11 normal cynomolgus monkey genomes.

The missense variants found in two PKD/PLD monkeys were predicted to result in amino acid changes in *PKD1, PKD2*, and *GANAB*. ADPKD is common nephropathy caused by mutations in either PKD1 or PKD2. In Mutation Database online: http://pkdb.mayo.edu, the probability of missense mutations in *PKD1* and *PKD2* genes is about 127/564 and 15/200 (22). However, according to the amino acid conservation analysis in different species where the mutation is located, and whether the mutation is in the key domain of the gene, the mutations of *PKD1*: (XM_015442355.1: c.1144G>C/ p. E382Q) and *GANAB*: (NM_001285075.1: c.2708T>C/p. V903A) were predicted to be likely pathogenic mutations.

Both the liver and kidneys of 010139 had different degrees of cystic changes, and numerous cysts of various sizes form in the liver. We attempted to find the cause of polycystic liver and polycystic kidney formation from WGS. We first focused on genes closely related to polycystic liver formation—such as *PRKCSH, PCLD, LPR5*, and *SEC63*—but did not find pathogenic mutations. The statistical data for PKD-related gene mutations in patients indicate that more than 30 gene mutations cause polycystic kidney and polycystic liver phenotypes. In patients, polycystic liver and polycystic kidney can occur either alone or in combination. However, the relationship between them has not been clarified, and many scholars speculate that polycystic kidney and polycystic liver are ciliary diseases, attributable to abnormal protein expression caused by related gene mutations that affect the primary cilia, causing ciliary dysfunction and eventually resulting in cystic effusion. Cilia are structures that are widely distributed on the surface of cells in various organs of the body; therefore, PKD-related gene mutations may be observed in multiple organs. Heterozygous non-synonymous SNVs in the *GANAB* and *PKD2* genes were identified by WGS of 010139's diseased tissues. Laarschot identified five novel *GANAB* variants in a cohort of 625 patients with ADPKD or ADPLD. Missense variant c.1835G>C p. (R612P) was predicted to disrupt the structure of the active site of the protein, likely reducing its activity (23). *PKD2* encodes a member of the polycystin protein family. The encoded protein is a multi-pass membrane protein that functions as a calcium-permeable cation channel and is involved in calcium transport and calcium signaling in renal epithelial cells. This protein interacts with polycystin 1, and they may be partners in a common signaling cascade involved in tubular morphogenesis. Mutations in this gene are associated with ADPLD with or without kidney cysts.

In the PKD-affected monkey 993563, it is speculated that *PKD1*: (XM_015442355.1: c.1144G>C/ p. E382Q) is a pathogenic mutation. The *PKD1* gene encodes a complete transmembrane protein that acts as a calcium-permeable cation channel and a regulator of intracellular calcium homeostasis. It is also involved in cell–cell and cell–matrix interactions and may modulate G-protein–coupled signal transduction pathways. It plays a role in renal tubular development, and mutations in this gene cause ADPKD and ADPLD.

Some limitations of this study need to be acknowledged. First, the tissue samples used to extract proteins and RNA should have been snap-frozen immediately at the time of collection. However, due to a lack of experience, tissue samples were not appropriately handled and preserved. Protein levels and RNA results could not be verified. Second, both monkeys in this study were sporadic cases and we could not trace their relatives, which resulted in insufficient data for genetic studies. Third, RDDC analysis and PKD mutation database comparison in humans are not sufficient; further functional studies are required to confirm the causality of the described variants. Additional genetic and environmental factors may also influence the manifestation of the disorder. In humans, the disease process in ADPKD patients with heterozygous mutations develops slowly, often with the gradual formation of large cysts over decades, and is relatively mild. The “two-hit” hypothesis explains this phenomenon. It is speculated that patients with heterozygous mutations initially carry a mutation only on one allele, but when the body is affected by other factors mutations on the other allele result, some cells in the diseased tissue may develop homozygous mutations, and cysts will gradually form at this time. One of the flaws of our method of tissue homogenization to obtain genomes is that the genome is completely mixed, with both normal and abnormal nuclei genomes present, making it difficult to identify whether cells have homozygous mutations. Although this hypothesis remains controversial, our analysis indicates that the heterozygous mutations we reported in the two cases of PKD- and PLD-affected cynomolgus monkeys were identical to those found in patients.

Identification and characterization of different genes and different variants have led to the development of mutation-based molecular diagnostics for ADPKD. Molecular diagnosis of hereditary diseases is particularly important for limiting disease progression and designing an effective intervention. It is possible to discover new pathogenic candidate genes and mutations through next-generation and *de novo* sequencing. Evidence indicates a relationship between the type of PKD gene mutation and the disease phenotype. Furthermore, in families affected by ADPKD, molecular testing may be helpful in evaluating potential kidney donors. Although related cases in many other animals, such as cats and deer, have been reported (24, 25), few specific types of gene mutations have been diagnosed. Genome sequence data from a larger number of wild animal species can provide references for the identification of putative PKD and PLD candidate genes and their associated mutations and thus contribute to a better understanding of the molecular pathogenesis of PKD in wild animals.

In this study, we diagnosed and reported two sporadic cases of PKD in non-human primate-aged cynomolgus monkeys in China, one of which was also accompanied by PLD. Their liver and kidney phenotypes were found to be highly similar to those of humans with the corresponding disease. We propose that the missense variants in *PKD1*: (XM_015442355.1: c.1144G>C/ p. E382Q) and *GANAB*: (NM_001285075.1: c.2708T>C/p. V903A) are most likely pathogenic for PKD/PLD based on WGS analysis results. The mutation sites we found were completely consistent with the sequence position of humans, which provides a reference for the molecular diagnosis of human PKD and PLD. Moreover, our findings provide a reference for the establishment of PKD animal models and future research on the pathogenesis of PKD and PLD.

## Data availability statement

ADPKD-affected monkeys' genome sequence reads from this study have been deposited in the Sequence Read Archive (SRA) database maintained by the National Center for Biotechnology Information (NCBI) (Accession Nos. SUB10161089, PRJNA752494, and SRP333006). WGS raw sequence data of wild-type monkeys used for comparative analysis in this article have been deposited in the Genome Sequence Archive (22, 26) in the BIG Data Center (Nucleic Acids Res 2018), Beijing Institute of Genomics (BIG), Chinese Academy of Sciences, under accession number CRA002684, and are publicly accessible at https://bigd.big.ac.cn/gsa.

## Ethics statement

The animal study was reviewed and approved by in accordance with the ethical standard guides for the Care and Use of Laboratory Animal in this study. Project number is KBI-K001116020-01,01, dated January 1, 2018 to December 31, 2019.

## Author contributions

XL, SL, YC, and RW designed the overall study. RW and RB completed experiments related to histology and whole-genome sequencing (WGS). BB and ZZ completed the analysis and interpretation of WGS data. RJ uploaded the WGS data to the NCBI database. FL and YZ performed veterinary and animal management. RW, BB, and FL wrote the original manuscript draft. XL, SL, and YC supervised the study, critically reviewed, and revised the manuscript. All authors reviewed the draft and approved the decision to submit it for publication.

## Supplementary material

Supplementary Figure S1 Macroscopic specimen of monkey 993563 and a healthy monkey. **(A)** The kidneys of PKD-affected monkey 993563. Several cysts filled with pale yellow tissue exudate on the surface (black arrow) of the renal cortex. **(B)** The liver visceral surface image of 993563, reddish brown with a soft texture and neat edges **(C, D)**. The kidneys and liver of a healthy monkey.

Supplementary Figure S2 Characterization of the identified functional candidate variant in PKD2 in 010139 PKD- and PLD-affected cynomolgus monkey. **(A)** Results of Sanger sequencing analysis of the forward strand of exon 1 of the *PKD2* gene in 010139. The position of the mutation is indicated with a gray bar. **(B)** Schematic representation of PKD2 indicating the c.C452T variant location in exon 1 (purple). Note that the cynomolgus monkey *PKD2* gene is annotated on the reverse complementary strand. **(C)** Multispecies protein alignment of the monkey PKD- and PLD-associated missense variant identified herein (Khaki).
