# Supplementary material for: Phenotypes and genetic etiology of spontaneous polycystic kidney and liver disease in cynomolgus monkey
Source: Front Vet Sci. 2023 Feb 16;10:1106016. doi: 10.3389/fvets.2023.1106016 (PMC9978152; doi:10.3389/fvets.2023.1106016)
Supplement: Supplementary file 1 [file Table_1.docx]

**Table S1. Reference values of hematologic and clinical chemistry parameters for male cynomolgus monkeys.**

| **Biochemical indicator** | **Value** | **Biochemical indicator** | **Value** |
| --- | --- | --- | --- |
| **Alanine aminotransferase (U/L)** | 35.08±13.33 | **Erythrocyte (×10^12^/L)** | 5.53±0.34 |
| **Alanine aminotransferase (U/L)** | 40.92±13.87 | **Leukocyte (×10^9^/L)** | 11.15±2.85 |
| **Alkaline phosphatase (U/L)** | 316.08±121.22 | **Platelet (×10^9^/L)** | 431.85±89.92 |
| **Total protein (g/L)** | 72.40±3.50 | **Hemoglobin (g/L)** | 132.08±5.77 |
| **Albumin (g/L)** | 42.20±2.75 | **Packed cell volume** | 0.40±0.02 |
| **A/g** | 1.40±0.12 | **Mean corpuscular volume (pg)** | 23.96±1.05 |
| **Cholesterol (mmol/L)** | 2.23±0.47 | **Mean corpuscular hemoglobin concentration (g/L)** | 333.85±4.91 |
| **Triglycerides (mmol/L)** | 0.50±0.25 | **Lymphocyte (%)** | 48.92±10.67 |
| **Creatinine (μmol/L)** | 101.85±13.85 | **Neutrophil (%)** | 50.54±10.20 |
| **Blood urea nitrogen (μmol/L)** | 8.42±1.60 | **Eosinophil (%)** | 0.23±0.60 |
| **glucose (U/L)** | 3.75±0.91 | **Basophil (%)** | 0.00±0.00 |
| **Ketone body (mmol/L)** | <0.6 | **Monocytes (%)** | 0.31±0.48 |
| **Na+** | 145.54±7.14 | **Prothrombin time (s)** | 10.54±0.42 |
| **K+** | 4.06±7.14 | **Reticulocyte (%)** | 0.83±0.40 |
| **Cl-** | 110.15±4.76 |  |  |
