# Supplementary figures and images for: Phenotypes and genetic etiology of spontaneous polycystic kidney and liver disease in cynomolgus monkey
Source: Front Vet Sci. 2023 Feb 16;10:1106016. doi: 10.3389/fvets.2023.1106016 (PMC9978152; doi:10.3389/fvets.2023.1106016)

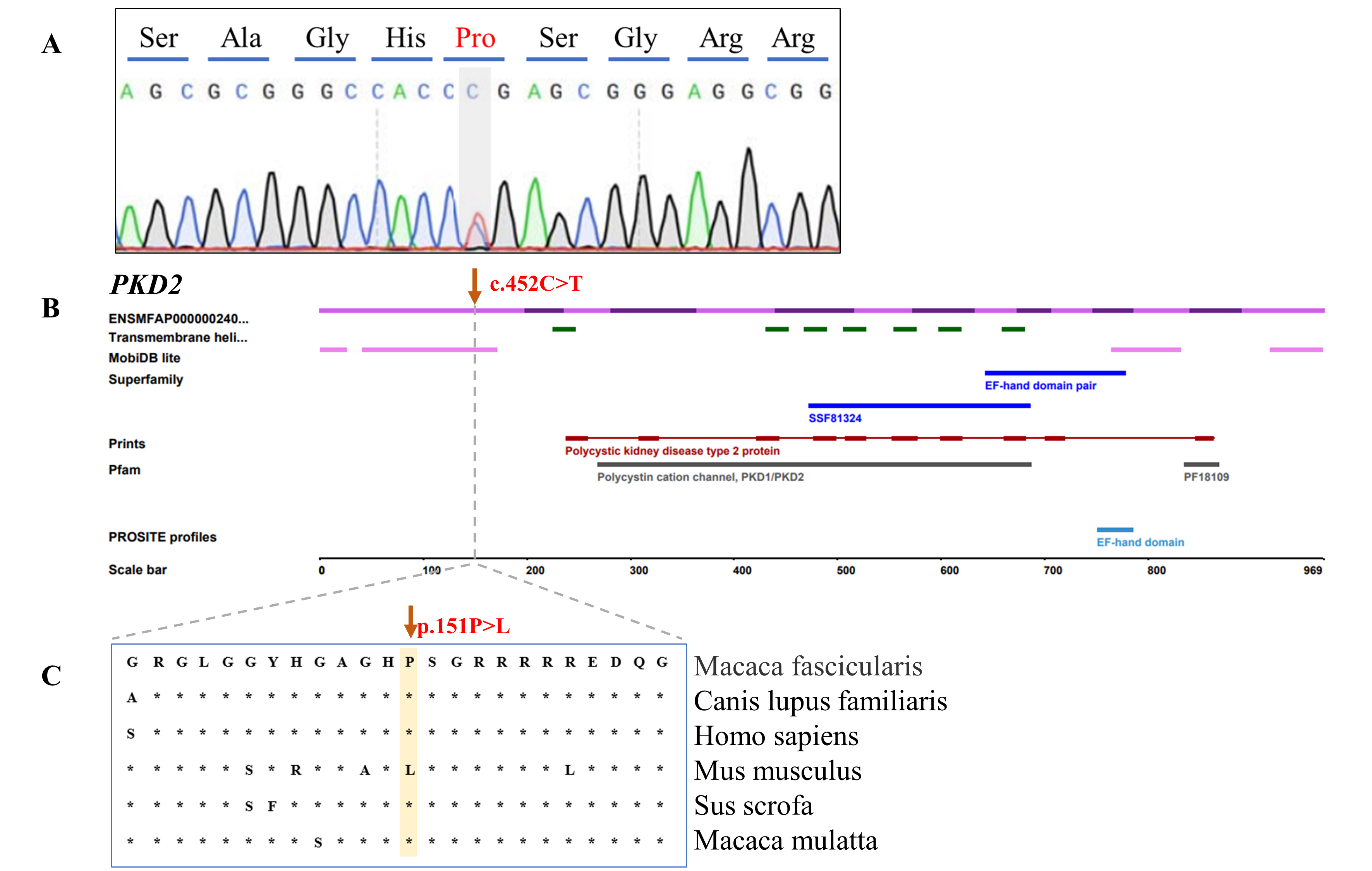

Supplement: Supplementary Figure S1 — Macroscopic specimen of monkey 993563 and a healthy monkey. (A) The kidneys of PKD-affected monkey 993563. Several cysts filled with pale yellow tissue exudate on the surface (black arrow) of the renal cortex. (B) The liver visceral surface image of 993563, reddish brown with a soft texture and neat edges (C, D). The kidneys and liver of a healthy monkey. [file Image_1.TIF]

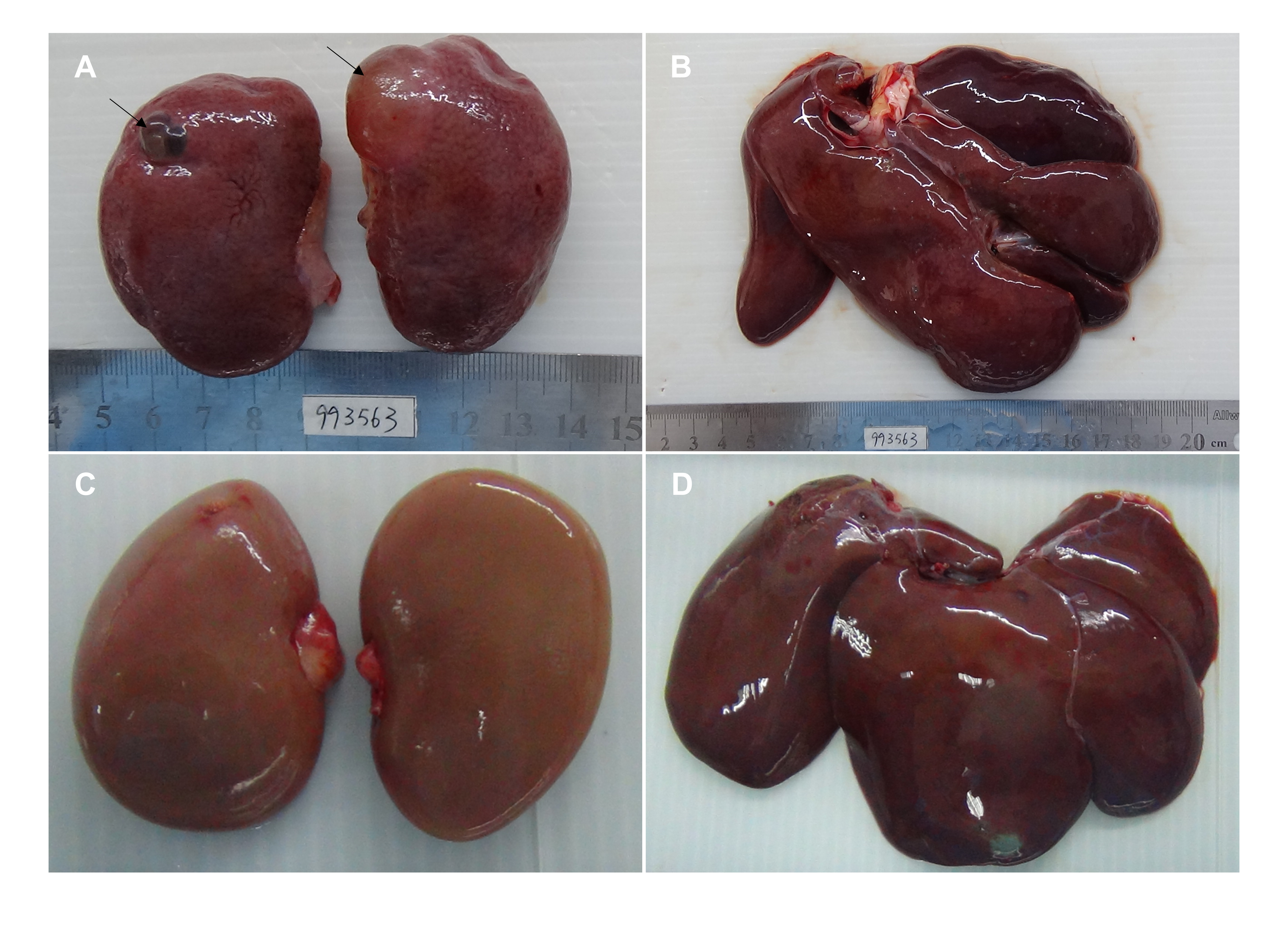

Supplement: Supplementary Figure S2 — Characterization of the identified functional candidate variant in PKD2 in 010139 PKD- and PLD-affected cynomolgus monkey. (A) Results of Sanger sequencing analysis of the forward strand of exon 1 of the PKD2 gene in 010139. The position of the mutation is indicated with a gray bar. (B) Schematic representation of PKD2 indicating the c.C452T variant location in exon 1 (purple). Note that the cynomolgus monkey PKD2 gene is annotated on the reverse complementary strand. (C) Multispecies protein alignment of the monkey PKD- and PLD-associated missense variant identified herein (Khaki). [file Image_2.TIF]
